# Gustav III’s risk assessment on coffee consumption; A medical history report

**Published:** 2017

**Authors:** Reza Afshari

**Affiliations:** 1 *Addiction Research Centre, Mashhad University of Medical Sciences, Mashhad, Iran*; 2 *Environmental Health Services, BC Centre for Disease Control, Vancouver, Canada*

**Keywords:** Coffee, Heart, Medical History

**Dear editor**

Coffee is one of the most commonly consumed drinks worldwide. For centuries, medicinal values and potential toxic effects related to coffee have been discussed. Consumption of coffee was not always welcome by physicians. However, it has been shown that even physicians commonly drink coffee as a stimulant (Giesinger et al., 2015 ▶). 

Recently, meta-analyses revealed that drinking coffee induces a dose-dependent risk reduction after acute myocardial infarction (Brown et al., 2016 ▶). In addition, more positive reports on drinking coffee are being published including 2016 surprising announcement by the Agency for Research on Cancer (IARC) (Brown et al., 2016 ▶) to discard its own previous report on cancerogenicity of coffee drinking in humans (IARC, 1991 ▶; Loomis et al., 2016 ▶). 

If coffee decreases the possibility of myocardial infarction and it is no longer harmful from a carcinogenicity point of view, it is the time to acknowledge Gustav III (1746-1792 CE) (Encyclopædia Britannica, 2016 ▶), the adventures king of Sweden’s, pioneer experiment on coffee safety as the first documented “randomized clinical trial” in medical history. In the same year that he was born, the “Misuse and Excesses Tea and Coffee Drinking Edict” was signed by his father, Adolph Frederick, and taxes were implemented on use with heavy fines and confiscation of cups and dishes for coffee drinkers if they do not confess they used this psychoactive intoxicant (Weinberg and Bealer, 2001 ▶). Later, a total ban was implemented in the country.

Gustav III, who has unfavorable views on coffee due to perceived health hazards, crowned in 1771 and ordered a human study on two identical twins. He commuted the death penalty for the crime committed by them to life imprisonment if they participate in his experiment. One twin agreed to drink three pots of coffee for the rest of his life, and the other one a similar amount of tea. Two prominent physicians were monitoring their health. Both physicians died before the experiment completed, one dying before the other. Gustav III himself was assassinated in 1792, while both twins lived healthily for a long time. Eventually, the tea consumer twin died at the age 83 years, and coffee won! The ban on coffee in Sweden was eventually lifted in 1820s (Cultural Heritage Group. 2013 ▶). 

It is plausible that high rate of coffee consumption (three pots per day) could had protected prisoner’s heart as its dose-dependent beneficial effect is now shown by Brown et al study. Medical history is full of surprises; authors may also have solved Gustave III’s two century old coffee mystery.

## Conflicts of Interest:

The author declares that he has no conflicts of interest to disclose. 
